# Value of CT-Based Radiomics in Predicating the Efficacy of Anti-HER2 Therapy for Patients With Liver Metastases From Breast Cancer

**DOI:** 10.3389/fonc.2022.852809

**Published:** 2022-04-07

**Authors:** Miao He, Yu Hu, Dongdong Wang, Meili Sun, Huijie Li, Peng Yan, Yingxu Meng, Ran Zhang, Li Li, Dexin Yu, Xiuwen Wang

**Affiliations:** ^1^Department of Oncology, Qilu Hospital, Cheeloo College of Medicine, Shandong University, Jinan, China; ^2^Department of Radiology, Qilu Hospital, Cheeloo College of Medicine, Shandong University, Jinan, China; ^3^Department of Oncology, Jinan Central Hospital, Cheeloo College of Medicine, Shandong University, Jinan, China; ^4^Department of Oncology, Central Hospital Affiliated to Shandong First Medical University, Jinan, China; ^5^Department of Oncology, Affiliated Hospital of Shandong University of Traditional Chinese Medicine, Jinan, China; ^6^Department of Comprehensive Section of Medical Affairs, Qilu Hospital, Cheeloo College of Medicine, Shandong University, Jinan, China; ^7^Huiying Medical Technology Co. Ltd, Beijing, China

**Keywords:** breast cancer, liver metastases, anti-HER2 therapy, radiomics, CT

## Abstract

**Objective:**

This study aims to assess the performance of machine learning (ML)-based contrast-enhanced CT radiomics analysis for predicating the efficacy of anti-HER2 therapy for patients with liver metastases from breast cancer.

**Methods:**

This retrospective study analyzed 83 patients with breast cancer liver metastases. Radiomics features were extracted from arterial phase, portal venous phase, and delayed phase images, respectively. The intraclass correlation coefficient (ICC) was calculated to quantify the reproducibility of features. The training and validation sets consisted of 58 and 25 cases. Variance threshold, SelectKBest, and LASSO logistic regression model were employed for feature selection. The ML classifiers were K-nearest-neighbor algorithm (KNN), support vector machine (SVM), XGBoost, RF, LR, and DT, and the performance of classifiers was evaluated by ROC analysis.

**Results:**

The SVM classifier had the highest score in portal venous phase. The results were as follows: The AUC value of the poor prognosis group in validation set was 0.865, the sensitivity was 0.77, and the specificity was 0.83. The AUC value of the good prognosis group in validation set was 0.865, the sensitivity was 0.83, and the specificity was 0.77. In arterial phase, the XGBoost classifier had the highest score. The AUC value of the poor prognosis group in validation set was 0.601, the sensitivity was 0.69, and the specificity was 0.38. The AUC value of the good prognosis group in validation set was 0.601, the sensitivity was 0.38, and the specificity was 0.69. The LR classifier had the highest score in delayed phase. The AUC value of poor prognosis group in validation set was 0.628, the sensitivity was 0.62, and the specificity was 0.67. The AUC value of the good prognosis group in validation set was 0.628, the sensitivity was 0.67, and the specificity was 0.62.

**Conclusion:**

Radiomics analysis represents a promising tool in predicating the efficacy of anti-HER2 therapy for patients with liver metastases from breast cancer. The ROI in portal venous phase is most suitable for predicting the efficacy of anti-HER2 therapy, and the SVM algorithm model has the best efficiency.

## Introduction

Breast cancer is the most common cancer in women all over the world, and its treatment has made substantial progress over the past years (1–3). Studies have shown that about 1/3 of breast cancer patients will have distant nonlymph node metastasis, once distant metastasis occurs, the 5-year survival rate will drop to 23% (4). The common sites of metastasis are the bone, lung, liver, and brain (5). About 50% of metastatic breast cancer (MBC) patients have liver metastasis, and the natural overall survival (OS) of these people is only 4–8 months (6). The liver metastasis of breast cancer [breast cancer liver metastasis (BCLM)] is one of the main causes of death in MBC patients. Although some progress has been made in chemotherapy, targeted therapy, and endocrine therapy for BCLM, the benefits of the current treatments are still limited; the average overall survival time of BCLM is only 3 years (7).

Breast cancer is also a malignancy with high heterogeneity at molecular level; there are significant differences in the treatment and prognosis of patients with different molecular subtypes of breast cancer (8). HER2-positive breast cancer is a subtype of breast cancer, which is associated with high invasiveness, high risk of recurrence, rapid progression, and poor prognosis and is an independent factor in poor prognosis of breast cancer patients (9–12). Fortunately, the use of anti-HER2 drugs has greatly improved the survival rate of these patients (13). As the first humanized monoclonal antibody targeting HER2, the advent of trastuzumab has affected the diagnosis and treatment mode of breast cancer (14–18). Clinical trials have also confirmed that other anti-HER2 drugs such as pyrotinib and lapatinib can significantly prolong the survival time of MBC patients (19–23). However, the efficacy of anti-HER2 drugs varies from person to person. Some patients who used anti-HER2 drugs can achieve an efficacy greater than the median progression-free survival (mPFS) and median overall survival (mOS) (19, 24, 25). Whereas, there were other patients with the same molecular typing who also used anti-HER2 drugs and failed to achieve the mean efficacy and lost their chance of survival (26, 27). Therefore, continued efforts to improve the efficiency of treatment are an imperative for management.

In recent years, artificial intelligence, especially the radiomics has developed rapidly. As an emerging technology to realize tumor segmentation, feature extraction, and model establishment, the radiomics can indirectly reflect the heterogeneity of tumors, find the correlation between quantitative data and pathological phenotype, and evaluate the whole tumor noninvasively, which has demonstrated predictive power for differential diagnosis and pathological classification, as well as the evaluation of response to treatment and prognosis (28, 29). However, a review of the literature published to date revealed no report on the predictive imaging features of anti-HER2 drugs for BCLM in connection with radiomics. Therefore, the aim of this study is to explore the feasibility of CT-based radiomics analysis by different ML classifiers for predicting the efficacy of anti-HER2 therapy in BCLM patients.

## Materials and Methods

### Patients

This retrospective study was approved by the Medical Ethics Committee of Qilu Hospital of Shandong University; patients’ informed consent was exempted after review by this ethics committee. The study population consisted of 83 patients, which were divided into the poor prognosis group and the good prognosis group, enrolled consecutively during the period from January 2011 to November 2021 in the Central Campus and East Campus of Qilu Hospital, Jinan Central Hospital, and Affiliated Hospital of Shandong University of Traditional Chinese Medicine. The poor prognosis group (PP group) included 42 cases (0 men, 42 women; mean age, 53.02 ± 9.64 years; median age, 53 years; range, 34–71 years). The good prognosis group (GP group) included 41 cases (0 men, 41 women; mean age, 52.59 ± 9.46 years; median age, 54 years; range, 32–78 years). All cases were pathologically confirmed by the primary or metastatic lesions (70 cases were pathologically confirmed by the primary lesions and 13 cases were pathologically confirmed by the metastatic lesions, including 7 cases were pathologically confirmed by liver metastases) and treated with anti-HER2 drugs after liver metastasis (trastuzumab 66, pyrotinib 11, lapatinib 6). The grouping criteria were based on the results of “H06489” trial that played a role in promoting trastuzumab as the first-line anti-HER2 drug for patients with MBC. This trial published in the New England Journal of Medicine in 2001 showed that for patients with HER2-positive MBC, the mPFS of chemotherapy plus trastuzumab was 7.4 months (25). A phase 3 clinical trial of MBC patients treated with trastuzumab and paclitaxel showed an mPFS of 12.5 months for pyrotinib plus capecitabine and 6.8 months for lapatinib plus capecitabine (20). According to the 2021 CSCO breast cancer guidelines, trastuzumab is the first choice of anti-HER2 drug for patients who had not used trastuzumab and who have used trastuzumab but eligible for reuse. A phase II clinical study of pyrotinib enrolled some patients who had not previously used trastuzumab, so the panel agreed that pyrotinib could also be applied to patients who have not failed trastuzumab therapy before (19). For patients with failed trastuzumab treatment, both pyrotinib and lapatinib can be used as first-line treatment for HER2-positive MBC. Therefore, in order to make the results more accurate and informative, we selected the mPFS of trastuzumab plus chemotherapy as the grouping criterion for this study; the enrolled patients were divided into two groups to compare their imaging features.

The inclusion criteria were as follows: (1) All patient’s pathology were obtained by operation or puncture and confirmed as HER2-positive breast cancer by immunohistochemical or FISH analysis; (2) Liver metastases were shown on CT images, and pathology or diagnostic imaging reports have confirmed liver metastases; and (3) Regularly using anti-HER2 drugs such as trastuzumab, pyrotinib, or lapatinib after finding liver metastases. Exclusion criteria included the following: (1) HER2 was negative for BCLM; (2) CT images had motion artifacts, poor image quality, different scanning conditions, and inconsistent layer thickness; and (3) PFS in patients treated with anti-HER2 drugs could not be determined.

### Image Data Acquisition

All contrast-enhanced CT images were obtained from SOMATOM Definition AS 64-detector row CT. The scanning range was from the top of the diaphragm to the inferior edge of the liver. The scanning conditions were as follows: tube voltage, 120 kV; automatic tube current; matrix, 512 × 512; scan layer thickness, 5 mm; and layer spacing, 5 mm. Lopromide was injected intravenously at the elbow with a flow rate of 3.0–3.5 ml/s and a dose of 1.0 ml/kg. Arterial phase, portal venous phase, and delayed phase scans were performed at 25–30, 60–70, and 120–180 s after contrast medium injection, and all the patients were able to cooperate with the examination normally. For patients with liver metastases found at first diagnosis, pathological results and CT images were obtained almost at the same time, and CT images were obtained almost at the same time as anti-HER2 drug therapy. For patients with liver metastases found after disease recurrence, pathology results were obtained before CT images, and the time interval between patient’s pathological results and the CT images vary from patient to patient. The acquisition of CT images was almost the same time as anti-HER2 drug therapy.

### Image Segmentation

**Figure 1:**
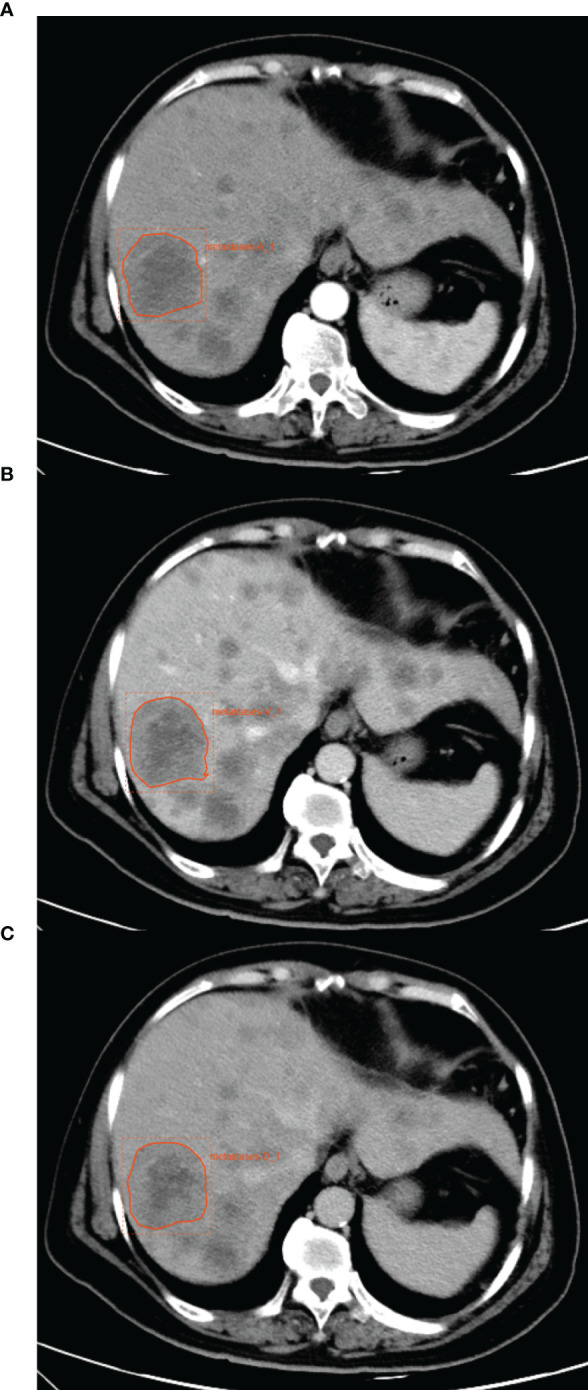
An example of manual segmentation of liver metastases from breast cancer. **(A)** The ROI in arterial phase. **(B)** The ROI in portal venous phase. **(C)** The ROI in delayed phase.

All these images were assessed and delineated in a double-blind manner by two radiologists with 5 and 10 years of experience, respectively, and following review was performed by the senior physician. If the difference was ≥5%, the latter would determine the boundary and redraw it. The maximum cross-sectional area of the largest liver metastases was uniformly selected as the VOI for outlining in all images. The grayscale normalization is carried out to reduce the influence of contrast and brightness changes. Finally, 246 ROI were segmented from the CT images of 83 patients (83 ROI in the arterial phase, 82 ROI in the portal venous phase, and 81 ROI in the delayed phase; one patient’s ROI in the venous phase and delayed phase and one patient’s ROI in the delayed phase were excluded from the enrollment because the thickness of the scanned layer was 1 mm, which did not meet our requirements), which were used for subject analysis. An example of the manual segmentation process is shown in **Figure 1**.

### Feature Extraction and Selection

A total of 1,409 quantitative radiomics features were extracted from CT images of the arterial phase, portal venous phase, and delayed phase, respectively, using the Radcloud platform (http://radcloud.cn/). These features can be grouped into three groups. Group 1 (first-order statistics) consisted of 126 descriptors that quantitatively delineate the distribution of voxel intensities within the CT image through commonly used and basic metrics. Group 2 (shape- and size-based features) contained 14 three-dimensional features that reflect the shape and size of the region. Calculated from gray-level run-length and gray-level co-occurrence texture matrices, 525 textural features that can quantify region heterogeneity differences were classified into group 3 (texture features). In addition, 14 kinds of filters such as exponent, logarithm, gradient, square root, lbp-2D, and wavelet (wavelet-LHL, wavelet-LHH, wavelet-HLL, wavelet-LLH, wavelet-HLH, wavelet-HHH, wavelet-HHL, wavelet-LLL) are used to filter the image, and the texture is analyzed on a finer scale.

To guarantee the robustness of the above features, an intraclass correlation coefficient (ICC) cutoff was set for test–retest analysis. The features with low repeatability were excluded from the follow-up analysis, and any features with ICC of less than 0.85 were discarded. To reduce the redundant features, the feature selection methods included the variance threshold, SelectKBest, and the least absolute shrinkage and selection operator (LASSO). For the variance threshold method, the threshold is 0.8, so that the eigenvalues of the variance smaller than 0.8 are removed. The SelectKBest method, which belongs to a single-variable feature selection method, used *p*-value to analyze the relationship between the features and the classification results; all the features with a *p*-value smaller than 0.05 will be used. For the LASSO model, L1 regularizer is used as the cost function, the error value of cross-validation is 5, and the maximum number of iterations is 1,000.

### Model Construction

The samples were randomly divided into training cohort (*n* = 58, 70%) and validation cohort (*n* = 25, 30%). To model the poor prognosis group and the good prognosis group, KNN, SVM, XGBoost, RF, LR, and DT classifiers were used. For KNN, the parameters were n_neighbors (5) and weights(uniform). For SVM, the parameters were kernel(rbf), C(1), gamma(auto), class_weight(balanced), decision_function_shape(ovr), and random_state(). For XGBoost, the parameters were Eta(0.3) and max_depth (6). For RF, the parameters were n_estimators (10) and class_weight(None). For LR, the parameters were penalty(L2), C(1), solver(liblinear), class_weight(None), multi_class(ovr), and random_state(). For DT, the parameters were splitter(best) and criterion(gini).

### Evaluation Index

The prediction performance was evaluated with a receiver operating characteristic (ROC) curve with the associated areas under the ROC curve (AUC), accuracy, sensitivity, and specificity. In order to estimate the generalization performance of a model, the models were validated in the test set. In addition, four indicators were used to evaluate the performance of the model, including precision (refers to the proportion of all predicted correct predictions in a sample), recall (actually predicted correct proportion in a sample), F1-score [F1-score = precision * recall *2/(precision + recall)], and support (the total number of samples involved). Using random grouping and taking the validation set results as the evaluation method for machine learning to evaluate the whole model’s classification accuracy. The average number of scores for each verification was taken to establish the score matrix, so as to select the appropriate ROI and select the best machine learning model.

### Statistical Analysis

Clinical data were analyzed with SPSS 24.0 (SPSS, Chicago, IL, USA). Age difference was tested by independent sample t-test, and *χ*^2^ test was used for hormone receptor status, HER2 status, physical status, previous use of chemotherapeutic drugs, recurrence, and metastasis status between the PP group and GP group. Through the linear combination of the selected features and the product of the corresponding weighting coefficients, the imaging labels of each patient were formed in turn, and the risk score of each patient based on each imaging tag was calculated. In the training set and verification set, the imaging features of the PP group and GP group were statistically analyzed, and the score matrix was established to compare and evaluate the results of different radiomics models. The ROC curve was used to evaluate the identification efficiency of the model. *p* < 0.05 was deemed to indicate statistical significance.

## Results

### Demographic Results

There was no significant difference in age, physical status, hormone receptor (HR) status, HER2 status, previous use of chemotherapeutic drugs, and recurrent and metastatic state between the PP group and GP group (**Table 1**).

**Table 1:** General status of subjects.

Group	PP	GP	*t*/*χ*^2^	*p*
**Number**	42	41	–	–
**Age**	53.02 ± 9.64	52.59 ± 9.46	−0.209	0.835
**HR**				
**Negative**	22	13	3.636	0.057
**Positive**	20	28		
**HER2**				
**3+**	32	31	0.004	0.951
**2+ FISH positive**	10	10		
**Physical status (ZPS)**				
**<2**	41	41	–	1
**≥2**	1	0		
**Previous use of chemotherapeutic drugs**				
**Paclitaxel**	32	31	0.004	0.951
**Anthracycline**	33	27	1.675	0.196
**Cyclophosphamide**	28	23	0.978	0.323
**Recurrence or metastasis within 12 months after (adjuvant) chemotherapy**				
**Yes**	16	12	0.725	0.696
**No**	16	18		
–	10	11		
**Liver metastasis was initially diagnosed**				
**Yes**	10	10	0.004	0.951
**No**	32	31		

### Feature Extraction and Screening Results

Take the portal venous phase as an example, the variance threshold method was used to select 362 features from 1,409 features (**SI Appendix, Figure S2A**), then with the select K best methods, we selected 9 features (**SI Appendix, Figure S2B**), finally, we selected 4 optimal features with the LASSO algorithm (**SI Appendix, Figures S2C–E**). Based on these 4 features and their regression coefficients, the radiomics score (Rad-score) formula was constructed as follows: **Rad** – **score** = **feature** * **coefficient** (**Table 2**). The feature extraction and screening results for the arterial phase and delay phase were described in **SI Appendix, Figures S1** and **S3** and **Tables S1** and **S2**.

**Table 2:** Description of the selected radiomics features with their associated feature group and filter in portal venous phase.

Radiomics feature	Radiomics class	Filter	Coefficient
Dependence variance	gldm	Wavelet-HLL	0.10773
Long run high gray-level emphasis	glrlm	Wavelet-HHL	−0.0914
Dependence variance	gldm	Wavelet-HLH	0.01859
Long run low gray-level emphasis	glrlm	Wavelet-LLH	0.08234

GLDM, gray-level dependence matrix; GLRLM, gray-level run-length matrix.

### Diagnostic Performance of Various Classifier Models

**Figure 2:**
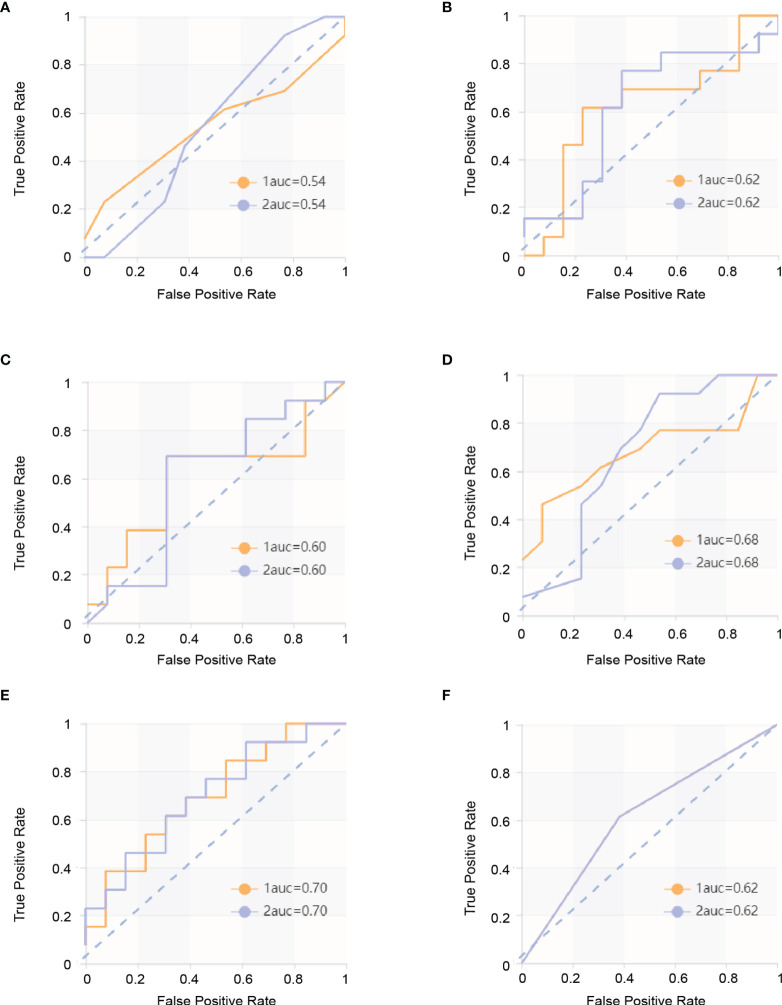
ROC curves in arterial phase. The yellow curve is the poor prognosis group (PP group), and the blue curve is the good prognosis group (GP group). **(A)** ROC curve of the KNN model in the validation set. The AUC were 0.544 in the PP group (sensitivity and specificity were 0.46 and 0.62, respectively) and 0.544 in the GP group (sensitivity and specificity were 0.46 and 0.62, respectively). **(B)** ROC curve of the SVM model in the validation set. The AUC were 0.621 in the PP group (sensitivity and specificity were 0.62 and 0.69, respectively) and 0.621 in the GP group (sensitivity and specificity were 0.69 and 0.62, respectively). **(C)** ROC curve of the XGBoost model in the validation set. The AUC were 0.601 in the PP group (sensitivity and specificity were 0.69 and 0.38, respectively) and 0.601 in the GP group (sensitivity and specificity were 0.38 and 0.69, respectively). **(D)** ROC curve of RF model in the validation set. The AUC were 0.680 in the PP group (sensitivity and specificity were 0.62 and 0.69, respectively) and 0.680 in the GP group (sensitivity and specificity were 0.69 and 0.62, respectively). **(E)** ROC curve of the LR model in the validation set. The AUC were 0.698 in the PP group (sensitivity and specificity were 0.62 and 0.62, respectively) and 0.698 in the GP group (sensitivity and specificity were 0.62 and 0.62, respectively). **(F)** ROC curve of the DT model in the validation set and ROC curve of the validation set. The AUC were 0.615 in the PP group (sensitivity and specificity were 0.62 and 0.62, respectively) and 0.615 in the GP group (sensitivity and specificity were 0.62 and 0.62, respectively).

**Figure 3:**
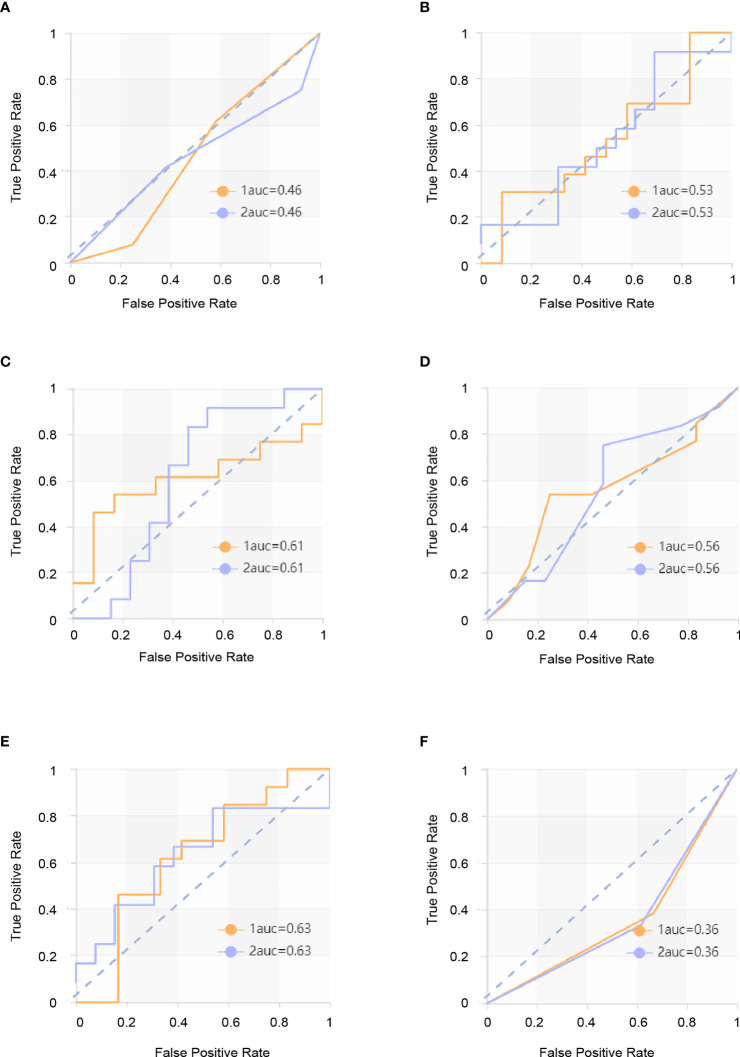
ROC curves in delayed phase. The yellow curve is the poor prognosis group (PP group), and the blue curve is the good prognosis group (GP group). **(A)** ROC curve of the KNN model in the validation set. The AUC were 0.462 in the PP group (sensitivity and specificity were 0.62 and 0.42, respectively) and 0.462 in the GP group (sensitivity and specificity were 0.42 and 0.62, respectively). **(B)** ROC curve of the SVM model in the validation set. The AUC were 0.532 in the PP group (sensitivity and specificity were 0.54 and 0.50) and 0.532 in the GP group (sensitivity and specificity were 0.50 and 0.54, respectively). **(C)** ROC curve of the XGBoost model in the validation set. The AUC were 0.609 in the PP group (sensitivity and specificity were 0.62 and 0.50, respectively) and 0.609 in the GP group (sensitivity and specificity were 0.50 and 0.62, respectively). **(D)** ROC curve of the RF model in the validation set. The AUC were 0.564 in the PP group (sensitivity and specificity were 0.54 and 0.58, respectively) and 0.564 in the GP group (sensitivity and specificity were 0.58 and 0.54, respectively). **(E)** ROC curve of the LR model in the validation set. The AUC were 0.628 in the PP group (sensitivity and specificity were 0.62 and 0.67, respectively) 0.578 in the GP group (sensitivity and specificity were 0.67 and 0.62, respectively). **(F)** ROC curve of the DT model in the validation set. The AUC were 0.359 in the PP group (sensitivity and specificity were 0.38 and 0.33, respectively) and 0.528 in the GP group (sensitivity and specificity were 0.33 and 0.38, respectively).

**Figure 4:**
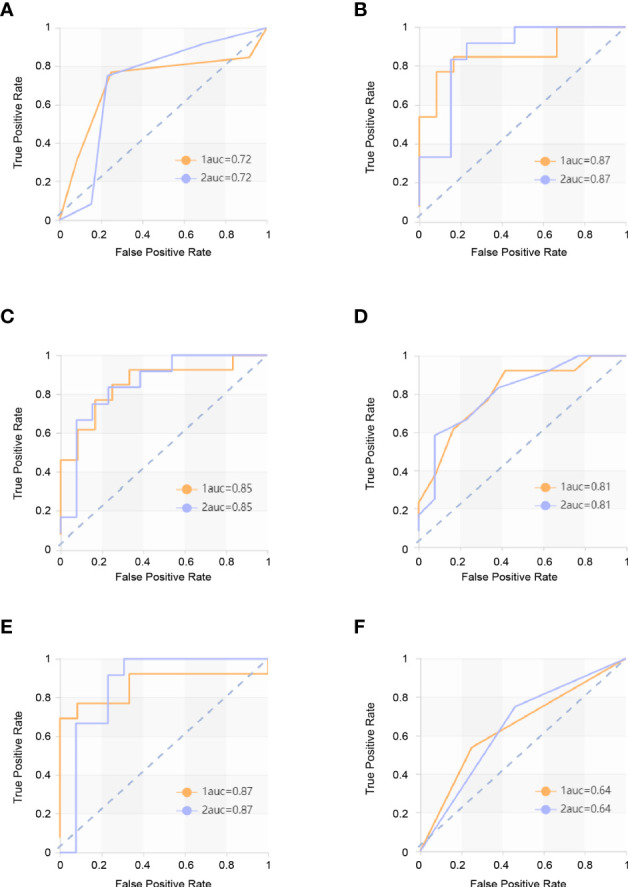
ROC curves in portal venous phase. The yellow curve is the poor prognosis group (PP group), and the blue curve is the good prognosis group (GP group). **(A)** ROC curve of the KNN model in the validation set. The AUC were 0.718 in the PP group (sensitivity and specificity were 0.77 and 0.75, respectively) and 0.718 in the GP group (sensitivity and specificity were 0.75 and 0.77, respectively. **(B)** ROC curve of the SVM model in the validation set. The AUC were 0.865 in the PP group (sensitivity and specificity were 0.77 and 0.83) and 0.865 in the GP group (sensitivity and specificity were 0.83 and 0.77, respectively). **(C)** ROC curve of the XGBoost model in the validation set. The AUC were 0.853 in the PP group (sensitivity and specificity were 0.85 and 0.67, respectively) and 0.853 in the GP group (sensitivity and specificity were 0.67 and 0.85, respectively). **(D)** ROC curve of the RF model in the validation set. The AUC were 0.808 in the PP group (sensitivity and specificity were 0.62 and 0.83, respectively) and 0.808 in the GP group (sensitivity and specificity were 0.83 and 0.62, respectively). **(E)** ROC curve of the LR model in the validation set. The AUC were 0.865 in the PP group (sensitivity and specificity were 0.77 and 0.75, respectively) and 0.865 in the GP group (sensitivity and specificity were 0.75 and 0.77, respectively). **(F)** ROC curve of the DT model in the validation set. The AUC were 0.644 in the PP group (sensitivity and specificity were 0.54 and 0.75, respectively) and 0.644 in GP group (sensitivity and specificity were 0.75 and 0.54, respectively).

The score matrix of the six classifiers in arterial phase, portal venous phase, and delayed phase are presented in **Table 3**. The results of the ROC curve analysis of all classifiers in the arterial phase and delayed phase are summarized in **Tables 4** and **5**, and the ROC curves are shown in **Figures 2** and **3**. When analyzing features in portal venous phase, all classifiers performed well, SVM classifier scored the highest, the AUC value of the PP group in the validation set was 0.865 (95% CI: 0.72~1.00; sensitivity, 0.77; specificity, 0.83), and the AUC value of the GP group in the validation set was 0.865 (95% CI: 0.72~1.00; sensitivity, 0.83; specificity, 0.77). The results of the ROC curve analysis of all classifiers in the portal venous phase are summarized in **Table 6**, and the ROC curves are shown in **Figure 4**. The four indicators of the portal venous phase (accuracy, recall, F1-score, support) are presented in **Table 7**.

**Table 3:** Results of score matrix of training sets and validation sets in arterial phase, portal venous phase, and delay phase.

Classifiers	Category	Arterial phase	Portal venous phase	Delayed phase
KNN	Training set	0.79	0.72	0.86
	Validation set	0.56	0.76	0.52
SVM	Training set	0.88	0.77	0.82
	Validation set	0.60	0.84	0.52
XGBoost	Training set	1	0.89	0.98
	Validation set	0.72	0.80	0.56
RF	Training set	1	0.98	0.98
	Validation set	0.48	0.72	0.48
LR	Training set	0.86	0.72	0.73
	Validation set	0.68	0.76	0.64
DT	Training set	1	1	1
	Validation set	0.52	0.64	0.36

**Table 4:** ROC results with six classifiers of validation set in arterial phase.

Classifiers	Category	AUC	95% CI	Sensitivity	Specificity
KNN	PP	0.544	0.35–0.74	0.62	0.46
	GP	0.544	0.35–0.74	0.46	0.62
SVM	PP	0.621	0.44–0.80	0.62	0.69
	GP	0.621	0.44–0.80	0.69	0.62
XGBoost	PP	0.601	0.42–0.79	0.69	0.38
	GP	0.601	0.42–0.79	0.38	0.69
RF	PP	0.680	0.50–0.86	0.62	0.69
	GP	0.680	0.51–0.89	0.69	0.62
LR	PP	0.698	0.51–0.89	0.62	0.62
	GP	0.698	0.49–0.91	0.62	0.62
DT	PP	0.615	0.42–0.81	0.62	0.62
	GP	0.615	0.42–0.81	0.62	0.62

**Table 5:** ROC s with six classifiers of validation set in delayed phase.

Classifiers	Category	AUC	95% CI	Sensitivity	Specificity
KNN	PP	0.462	0.26–0.67	0.62	0.42
	GP	0.462	0.26–0.67	0.42	0.62
SVM	PP	0.532	0.33–0.74	0.54	0.50
	GP	0.532	0.33–0.74	0.50	0.54
XGBoost	PP	0.609	0.40–0.82	0.62	0.50
	GP	0.609	0.40–0.82	0.50	0.62
RF	PP	0.564	0.36–0.76	0.54	0.58
	GP	0.564	0.36–0.76	0.58	0.54
LR	PP	0.628	0.43–0.82	0.62	0.67
	GP	0.628	0.43–0.82	0.67	0.62
DT	PP	0.359	0.17–0.55	0.38	0.33
	GP	0.359	0.17–0.55	0.33	0.38

**Table 6:** ROC results with six classifiers of validation set in portal venous phase.

Classifiers	Category	AUC	95% CI	Sensitivity	Specificity
KNN	PP	0.718	0.55–0.89	0.77	0.75
	GP	0.718	0.55–0.89	0.75	0.77
SVM	PP	0.865	0.72–1.00	0.77	0.83
	GP	0.865	0.72–1.00	0.83	0.77
XGBoost	PP	0.853	0.68–1.00	0.85	0.67
	GP	0.853	0.68–1.00	0.67	0.85
RF	PP	0.808	0.64–0.98	0.62	0.83
	GP	0.808	0.64–0.98	0.83	0.62
LR	PP	0.865	0.70–1.00	0.77	0.75
	GP	0.865	0.70–1.00	0.75	0.77
DT	PP	0.644	0.45–0.84	0.54	0.75
	GP	0.644	0.45–0.84	0.75	0.54

**Table 7:** The results of four indicators—precision, recall, F1-score, and support in validation set.

	Indicators	KNN	SVM	XGBoost	RF	LR	DT
PP	Precision	0.77	0.83	0.73	0.80	0.77	0.70
	Recall	0.77	0.77	0.85	0.62	0.77	0.54
	F1-score	0.77	0.80	0.79	0.70	0.77	0.61
	Support	13	13	13	13	13	13
GP	Precision	0.75	0.77	0.80	0.67	0.75	0.60
	Recall	0.75	0.83	0.67	0.83	0.75	0.75
	F1-score	0.75	0.80	0.73	0.74	0.75	0.67
	Support	12	12	12	12	12	12

## Disscusion

In this study, we evaluated the performance of a quantitative CT radiomics analysis combined with different ML-based classification schemes for predicting the efficacy of anti-HER2 therapy for BCLM patients. There are a variety of machine learning methods that can be used to build radiomics models, and they have their own advantages for different tasks. In this study, we used six commonly used classifier models (KNN, SVM, XGBoost, RF, LR, and DT) to evaluate the performance for discriminating the PP group from the GP group with three kinds of ROI. We found that the radiomics classifer demonstrated low performance for differentiation when using ROI of the arterial phase and delayed phase. The ROI of the portal venous phase performed better for each classifier and demonstrated high performance. Our preliminary results show that classifiers trained with ROI of the portal venous phase have better performance on discrimination between the PP group and GP group with significantly higher AUC than the ROI of the arterial phase and delayed phase in the validation set and in all patients.

Distant metastasis is the main lethal cause for advanced breast cancer patients (6, 30). According to the NCCN guidelines and the routine diagnosis and treatment in China, patients with HER2-positive MBC should be treated with continuous anti-HER2 therapy (31, 32). However, not every HER2-positive MBC patient can obtain satisfactory outcomes from anti-HER2 therapy clinically. Therefore, accurate prediction of the efficacy of anti-HER2 therapy for patients with advanced breast cancer will help guide treatment and potentially resulting in greater survival benefits for patients.

Of the four significant radiomics features in the venous phase finally screened out by the LASSO algorithm, we found that the texture feature of the PP group was different from the GP group. The main differences between them in terms of radiomics are gray-level run-length matrix and gray-level dependence.

The center of liver metastasis is usually hypodense, presenting a concentric circular or double contour structure, and edge enhancement is due to the hoof tissue at the margin of the tumor, inflammatory cell invasion, and vascular proliferation (33). Frederick et al. found significantly more liver metastases on portal venous-dominant phase than in the arterial-dominant phase or unenhanced images. This conclusion was further supported by the results of a larger study from the same group, in which the addition of unenhanced or arterial-dominant phase imaging did not reveal substantially more metastases compared with portal venous-dominant phase imaging alone (34–36). Although the liver metastases are significantly enhanced in the arterial phase, the degree of enhancement is significantly improved in the portal venous phase, which is presumably due to the contrast-enhanced arterial blood diffusing into the tumor neovasculature and tumor interstices during the portal venous phase. In malignant tumors, there are a large number of nourish blood vessels with tortuous and irregular paths, endothelial cells, and arteriovenous fistulas, and there are also microscopic cancer thrombi in some of the tumors, which increase the contrast medium’s access to the vascular contrast area but increase the resistance at the same time, thus making the enhancement in the portal phase clearer and longer and better showing its imaging features. The portal venous phase is also usually the best choice for showing the additional features of liver tumors as well as vascular anatomical and pathological conditions (37). Signal mediated by HER2 receptor can promote the secretion of vascular endothelial growth factor (VEGF), the tumor-associated angiogenesis, and the growth of tumor (38–40). Therefore, we believe that the liver metastases with stronger HER2 expression have richer tumor neovasculature, stronger and longer portal venous phase enhancement, which can better demonstrate the imaging characteristics of liver metastases. In other words, the portal venous phase better demonstrates the imaging features of liver metastases associated with HER2 expression and the efficacy of anti-HER2 drugs.

This study has several limitations: (1) Manual segmentation inevitably leads to subjective errors. (2) The present study is a retrospective study with a small sample size, additional data should be used to confirm the results in order to make the extrapolation of the model more credible.

In conclusion, the current study showed the feasibility of CT-based radiomics in predicating the efficacy of anti-HER2 therapy for BCLM, and the SVM algorithm model in the portal venous phase of contrast-enhanced CT has the best efficiency. The additional information provided by CT-based radiomics can help clinicians predict the therapeutic effect of anti-HER2 therapy and formulate management decisions, promoting the development of personalized precision therapy.

## Data Availability Statement

The original contributions presented in the study are included in the article/**Supplementary Material**. Further inquiries can be directed to the corresponding author.

## Ethics Statement

The studies involving human participants were reviewed and approved by the Medical Ethics Committee of Qilu Hospital of Shandong University. Written informed consent for participation was not required for this study in accordance with the national legislation and the institutional requirements.

## Author Contributions

YH conceived and designed the study; XW conducted the project. MS, HL, PY, LL, MH, YH and YM performed manuscript preparation, DW, DY and RZ analyzed the data. All authors discussed and interpreted the results. MH wrote this article, MH and YH revised it critically for important intellectual content. The article was subsequently reviewed and approved by all authors. All authors listed have made a substantial, direct, and intellectual contribution to the work and approved it for publication.

## Funding

This work was supported by grants from the National Natural Science Foundation of China (No. 81874044), the National Natural Science Foundation of China (grant number: 81600092), the Shandong Provincial Natural Science Foundation (No. ZR2019MH050), and China Anti-Cancer Association-Her2 Target Project of China Scientific Research Foundation in 2020-2021 (serial number:17).

## Conflict of Interest

Author RZ was employed by Huiying Medical Technology Co. Ltd., Beijing, China.

The remaining authors declare that the research was conducted in the absence of any commercial or financial relationships that could be construed as a potential conflict of interest.

## Publisher’s Note

All claims expressed in this article are solely those of the authors and do not necessarily represent those of their affiliated organizations, or those of the publisher, the editors and the reviewers. Any product that may be evaluated in this article, or claim that may be made by its manufacturer, is not guaranteed or endorsed by the publisher.
