# Supplementary material for: Value of CT-Based Radiomics in Predicating the Efficacy of Anti-HER2 Therapy for Patients With Liver Metastases From Breast Cancer
Source: Front Oncol. 2022 Apr 7;12:852809. doi: 10.3389/fonc.2022.852809 (PMC9021495; doi:10.3389/fonc.2022.852809)
Supplement: Supplementary file 1 [file DataSheet_1.docx]

**Supplementary Table 1.** Description of the selected radiomic features with their associated feature group and filter in arterial phase.

| Radiomic feature | Radiomic class | Filter | Coefficient |
| --- | --- | --- | --- |
| Total Energy | firstorder | wavelet-HLH | -0.06103 |
| Dependence Variance | gldm | wavelet-HLH | 0.09208 |
| Large Dependence Emphasis | gldm | wavelet-HHH | -0.04604 |
| Zone Entropy | glszm | wavelet-LHH | -0.02479 |

**Supplementary Table 2.** Description of the selected radiomic features with their associated feature group and filter in delay phase.

| Radiomic feature | Radiomic class | Filter | Coefficient |
| --- | --- | --- | --- |
| Long Run High Gray Level Emphasis | glrlm | wavelet-HLL | 0.15884 |
| Zone Entropy | glszm | wavelet-HHL | -0.08014 |
| Long Run Emphasis | glrlm | wavelet-HLL | 0.00144 |
| High Gray Level Emphasis | gldm | wavelet-LLH | -0.06401 |
| Kurtosis | firstorder | gradient | -0.05353 |
| Run Length Non Uniformity | glrlm | original | -0.01843 |
| Kurtosis | firstorder | wavelet-LHL | -0.01862 |

**
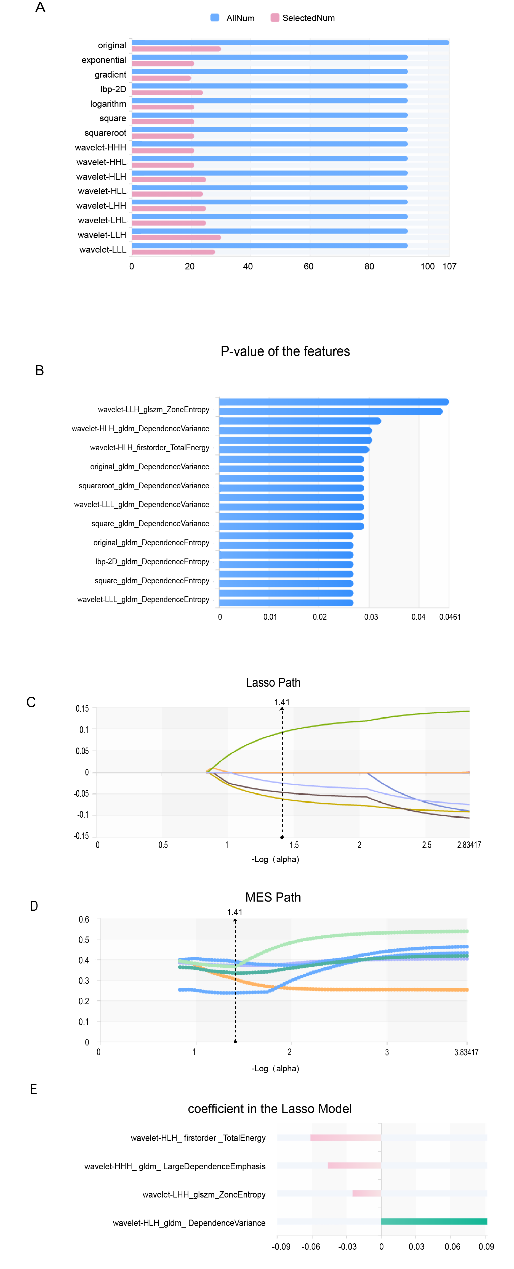
**

**Supplementary Figure 1.** The feature extraction and screening results for arterial phase. **(A)** Variance threshold on feature select. We used variance threshold methods to select radiomics features (variance threshold = 0.8), the blue bar represents all the extracted radiomics features numbers, the pink bar represents the radiomics features numbers filtered by the variance threshold method, and the ordinate is 15 filtering methods. We selected 357 features from 1409 features. **(B)**Select K best on feature select. The abscissa is the p-value of the feature. We used Select K best methods to further select radiomics features, and we selected 22 features. **(C)** Lasso althorithm on feature select. Lasso path, the abscissa is the log value of α, and the ordinate represents the coefficient of feature. (**D)** MSE path, the abscissa is the log value of α, and the ordinate represents the mean square error. **(E)** Coefficient in the Lasso model, the abscissa represents the regression coefficient and the ordinate represents the selected features. Using Lasso model, 4 features which are correspond to the optimal alpha value were selected.


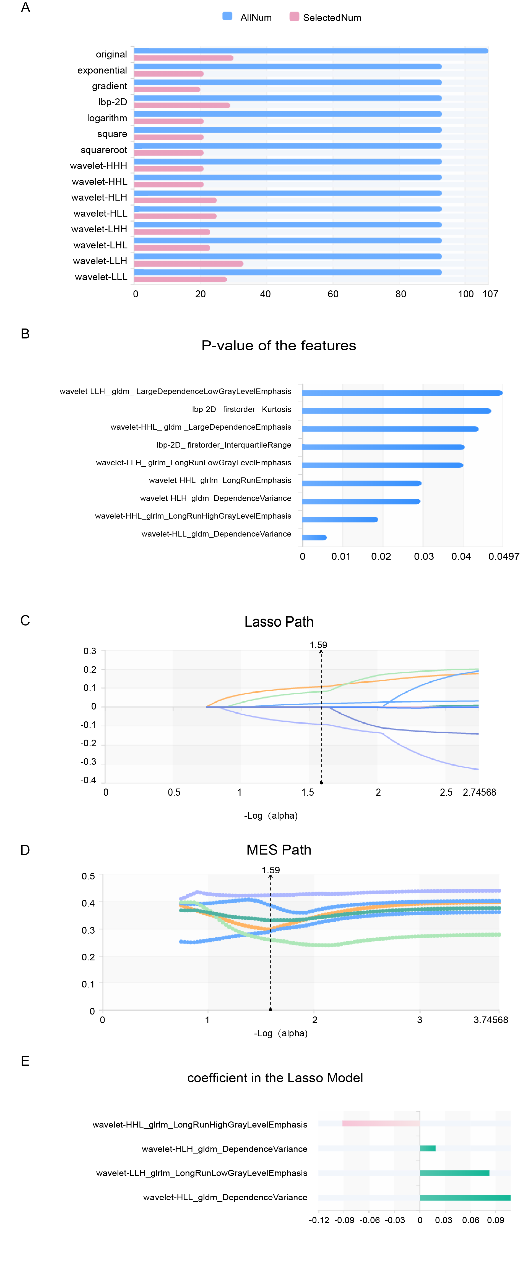


**Supplementary Figure 2.** The feature extraction and screening results for portal venous phase. **(A)** Variance threshold on feature select. We used variance threshold methods to select radiomics features (variance threshold = 0.8), the blue bar represents all the extracted radiomics features numbers, the pink bar represents the radiomics features numbers filtered by the variance threshold method, and the ordinate is 15 filtering methods. We selected 362 features from 1409 features. **(B)** Select K best on feature select. The abscissa is the p-value of the feature. We used Select K best methods to further select radiomics features, and we selected 9 features. **(C)** Lasso althorithm on feature select. Lasso path, the abscissa is the log value of α, and the ordinate represents the coefficient of feature. (**D)** MSE path, the abscissa is the log value of α, and the ordinate represents the mean square error. **(E)** Coefficient in the Lasso model, the abscissa represents the regression coefficient and the ordinate represents the selected features. Using Lasso model, 4 features which are correspond to the optimal alpha value were selected.


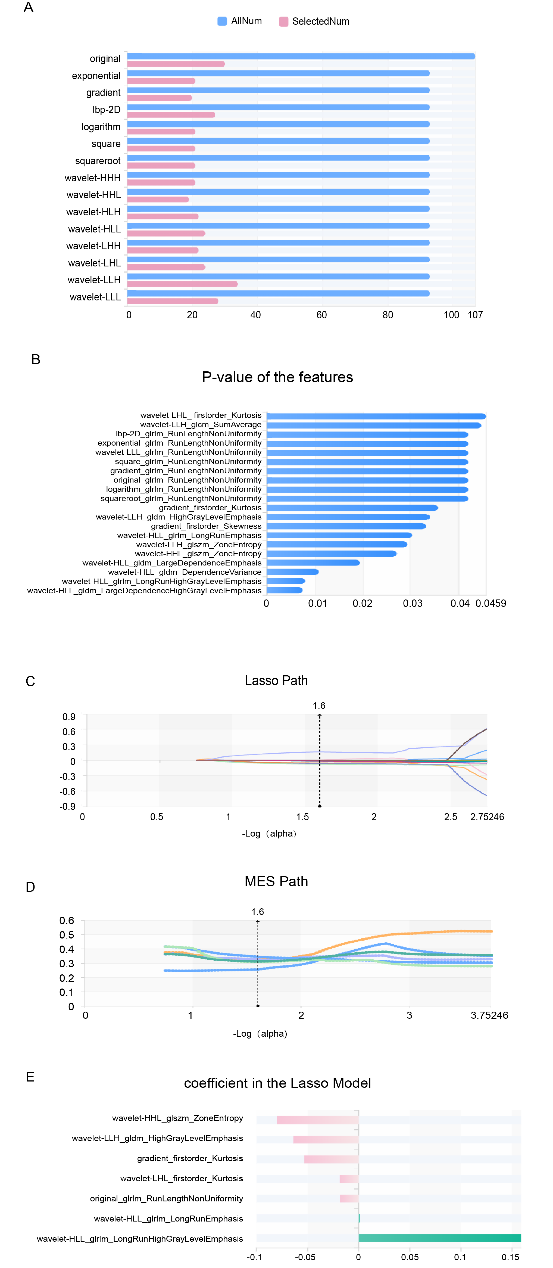


**Supplementary Figure 3.**The feature extraction and screening results for delay phase. **(A)** Variance threshold on feature select. We used variance threshold methods to select radiomics features (variance threshold = 0.8), the blue bar represents all the extracted radiomics features numbers, the pink bar represents the radiomics features numbers filtered by the variance threshold method, and the ordinate is 15 filtering methods. We selected 355 features from 1409 features. **(B)** Select K best on feature select. The abscissa is the p-value of the feature. We used Select K best methods to further select radiomics features, and we selected 20 features. **(C)** Lasso althorithm on feature select. Lasso path, the abscissa is the log value of α, and the ordinate represents the coefficient of feature. (**D)** MSE path, the abscissa is the log value of α, and the ordinate represents the mean square error. **(E)** Coefficient in the Lasso model, the abscissa represents the regression coefficient and the ordinate represents the selected features. Using Lasso model, 7 features which are correspond to the optimal alpha value were selected.
